# Performance of the high-dimensional propensity score in adjusting for unmeasured confounders

**DOI:** 10.1007/s00228-016-2118-x

**Published:** 2016-08-30

**Authors:** Jason R Guertin, Elham Rahme, Jacques LeLorier

**Affiliations:** 1 Pharmacoeconomic and Pharmacoepidemiology unit, Research Center of the Centre hospitalier de l’Université de Montréal, Tour St-Antoine, 850 St-Denis, Montreal, QC H2X 0A9 Canada; 2 Programs for Assessment of Technology in Health, 43 Charlton Avenue East, 2nd floor, Hamilton, ON L8N 1Y3 Canada; 3 Department of Clinical Epidemiology and Biostatistics, McMaster University, Hamilton, ON Canada; 4 Division of Clinical Epidemiology, Research Institute of the McGill University Health Centre, 1650, Cedar Ave, Montréal, QC H3G 1A4 Canada; 5 Department of Medicine, McGill University, Montreal, Canada

**Keywords:** Confounding by indication, High-dimensional propensity scores, Unmeasured confounders, Omitted confounders

## Abstract

**Purpose:**

High-dimensional propensity scores (hdPS) can adjust for measured confounders, but it remains unclear how well it can adjust for unmeasured confounders. Our goal was to identify if the hdPS method could adjust for confounders which were hidden to the hdPS algorithm.

**Method:**

The hdPS algorithm was used to estimate two hdPS; the first version (hdPS-1) was estimated using data provided by 6 data dimensions and the second version (hdPS-2) was estimated using data provided from only two of the 6 data dimensions. Two matched sub-cohorts were created by matching one patient initiated on a high-dose statin to one patient initiated on a low-dose statin based on either hdPS-1 (*Matched hdPS Full Info Sub-Cohort*) or hdPS-2 (*Matched hdPS Hidden Info Sub-Cohort*). Performances of both hdPS were compared by means of the absolute standardized differences (ASDD) regarding 18 characteristics (data on seven of the 18 characteristics were hidden to the hdPS algorithm when estimating the hdPS-2).

**Results:**

Eight out of the 18 characteristics were shown to be unbalanced within the unmatched cohort. Matching on either hdPS achieved adequate balance (i.e., ASDD <0.1) on all 18 characteristics.

**Conclusion:**

Our results indicate that the hdPS method was able to adjust for hidden confounders supporting the claim that the hdPS method can adjust for at least some unmeasured confounders.

## Introduction

The high-dimensional propensity score (hdPS) has been used in different contexts and within multiple databases for the control of confounding by indication and it has been shown to be at least equivalent and potentially superior to the propensity score in this regard [1–7]. Superiority of the hdPS is generally attributed to the greater number of covariates drawn from the database to include in the final hdPS model [5]. However, the performance of the hdPS has not been assessed when information regarding some of these potential confounders within the examined database is limited.

Our aim was to assess the impact of limited information regarding potential confounders on the performance of the hdPS. To achieve this goal, we compared the performance of the hdPS in a scenario where the algorithm had full access to all of the data contained within a database to its performance in a scenario where only partial data were available to the algorithm.

The administrative database situation in Quebec, Canada provides an interesting setting in which to examine this issue. There are two distinct sets of medico-administrative databases available in Quebec; the *Régie de l’assurance maladie du Québec* (RAMQ) databases (physician and pharmacists billing data) and the *Maintenance et Exploitation des Données pour l’Étude de la Clientèle Hospitalière* (MED-ECHO) databases (hospital discharge data). RAMQ and MED-ECHO data may overlap. However, they differ on their source of information (e.g., only the RAMQ databases provide outpatient data) and may be more detailed in specific areas (e.g., the MED-ECHO databases provide more detailed and more precise information regarding patients’ entry date/discharge date and on in-hospital diagnoses and therapeutic and diagnostic procedures) [8–10].

To test the performance of the hdPS under conditions of limited information regarding potential confounders, we examined the association between the risk of diabetes and exposure to high versus low statin doses [7, 11–16]. Assessing this association in a Quebec incident statin user population may be hindered by the presence of confounding by indication since patients started on a higher statin dose have been shown to be sicker and at higher risk for diabetes than those started on a lower dose [7].

We compared the performance of the hdPS within two scenarios: (1) the algorithm used in the hdPS estimation had full access to all the data provided by both the MED-ECHO and RAMQ databases, and (2) the algorithm had only access to the data provided by the MED-ECHO databases.

One of the uses of hdPS is to select a matched sub-cohort from the main cohort (all patients initiated on statins) where the characteristics of patients who received treatment A (high dose statins) are similar to the characteristics of patients who received treatment B (low-dose statins) [5]. That is, we assessed the performance of the hdPS on its ability to select a balanced sub-cohort when it is used as a matching criterion [7, 17–21]. The performance of the restricted information hdPS was assessed by comparing the balance achieved with this method to the balance achieved when all information was available to the algorithm.

## Methods

### Data sources

The different data sources used within this study have been described elsewhere [7]. Briefly, we obtained data on a cohort of 800,551 new statin users from RAMQ. For this study, we used data from both the RAMQ databases (i.e., demographic database, medical services, and claims database and pharmaceutical database) and from the MED-ECHO databases (i.e., hospitalization—description database, hospitalization—diagnoses database, and hospitalization—intervention database). Patient records were linked across all databases by use of a unique identification number which was encrypted to protect patient confidentiality. Access to data was granted by the *Commission d’accès à l’information* and the protocol was approved by the *Centre hospitalier de l’Université de Montréal*’s ethics’ committee.

### Full cohort

The *Full Cohort* used within this study has been described elsewhere [7]. Briefly, it was comprised of 404,129 patients newly initiated on a statin (either simvastatin, lovastatin, pravastatin, fluvastatin, atorvastatin, or rosuvastatin) between January 1st, 1998 and December 31st, 2010. Patients were defined as having been newly initiated on a statin if they did not receive any statin dispensation in the year prior to the date of first statin dispensation (hereby defined as the cohort entry date).

### Identification of exposure group

All patients were categorized into two groups based on the strength of the daily statin dose of their first statin dispensation [12]. Patients initiated on a daily dose of ≥10 mg of rosuvastatin, ≥20 mg of atorvastatin or ≥40 mg of simvastatin formed the high dose group and the remaining patients formed the low dose group.

### Identification of the study outcome

Onset of diabetes within 2 years follow-up was used as our study outcome. Patients were defined as cases if they received either a dispensation of a drug used in the treatment of diabetes (WHO ATC A10) or a diagnosis of diabetes (ICD-9 code: 250.x; ICD-10 codes: E10.x—E14.x) within the 2 years following the cohort entry date; all other patients were considered to be diabetes-free.

### High-dimensional propensity score method

Two distinct hdPS models were created and resulting hdPS were calculated for all patients included in the *Full Cohort*. Detailed description of the hdPS method can be found elsewhere [5]. Both models were created using the default setting of the SAS hdPS macro v.1 [22].

Six potential data dimensions were defined using the data collected from the year prior to the cohort entry date: (1) drugs dispensed in an outpatient setting, (2) physician claims for procedures codes, (3) physician claims for diagnostic codes, (4) specialty of the physician providing care, (5) hospitalization discharge data for inpatient procedure codes, and (6) hospitalization discharge data for inpatient diagnostic code.

### Full information model

The first hdPS model (hereby defined as *hdPS full info* model) was created by selecting the top 500 covariates, as assessed by the hdPS algorithm, contained within all 6 data dimensions. In addition to these 500 covariates, the following known confounders were forced within the *hdPS full info* model: [12] patients’ sex, age, poverty level status (yes versus no) at the cohort entry date, year of entry within the cohort (as a categorical variable), and ≥1 hospitalization, ≥5 outpatient visits, ≥5 distinct drugs dispensed to the patient, all within the year prior to the cohort entry date. The resulting *hdPS full info* model was used to estimate each patient’s hdPS-1.

### Hidden information model

The second hdPS model (hereby defined as the *hdPS hidden info* model) was created by selecting the top 500 covariates, as assessed by the hdPS algorithm, contained within the 2 data dimensions provided from the MED-ECHO databases since it was believed a priori that it would contain less potential covariates, therefore increasing the risk of unmeasured confounding (the 4 data dimensions provided by RAMQ were hidden to the algorithm). In addition to these 500 variables, the following covariates were forced within the *hdPS hidden info* model: patients’ sex, age, and poverty level status (yes versus no) at the cohort entry date, the year of entry within the cohort (as a categorical variable) and ≥1 hospitalization in the year prior to the cohort entry date. Within this model, hospitalization status (≥1 hospitalization yes vs no) was assessed solely from data available within the MED-ECHO databases. Outpatient medical resource utilization and outpatient drug dispensation covariates, forced within the previous model, were excluded from this list since they were based on information solely available within the RAMQ databases. The resulting *hdPS hidden info* model was used to estimate each patient’s hdPS-2.

### Creation of the matched sub-cohorts

Trimming was performed and patients located within non-overlapping regions of the hdPS-1 distribution were excluded [23–25], all other patients were eligible for inclusion within the *Matched hdPS Full Info Sub-Cohort*. Low dose controls were found for patients initiated on a high dose using a greedy, nearest neighbor 1:1 matching algorithm. Matching occurred if the difference in the logit of hdPS-1 between the nearest neighbors was within a caliper width equal to 0.2 times the SD of the logit of the hdPS-1 [26]. Patients selected by the matching algorithm were included within the *Matched hdPS Full Info Sub-Cohort.* These two steps were reproduced using hdPS-2 in order to create the *Matched hdPS Hidden Info Sub-Cohort*.

### Statistical analyses

Patients’ baseline characteristics within both sub-cohorts were assessed using the information provided from the full database. Absolute standardized differences (ASDD) were used to compare patients’ baseline characteristics between patients included in the high dose group versus those included in the low dose group within both matched sub-cohorts [19, 21]. ASDD <0.1 are generally assumed to indicate good balance between groups [21, 27].

Discrete data are presented in absolute values and percentages and continuous data are presented as mean (± SD). All statistics were performed using SAS version 9.3 (Cary, North Carolina).

## Results

### Description of the full cohort

**Fig. 1:**
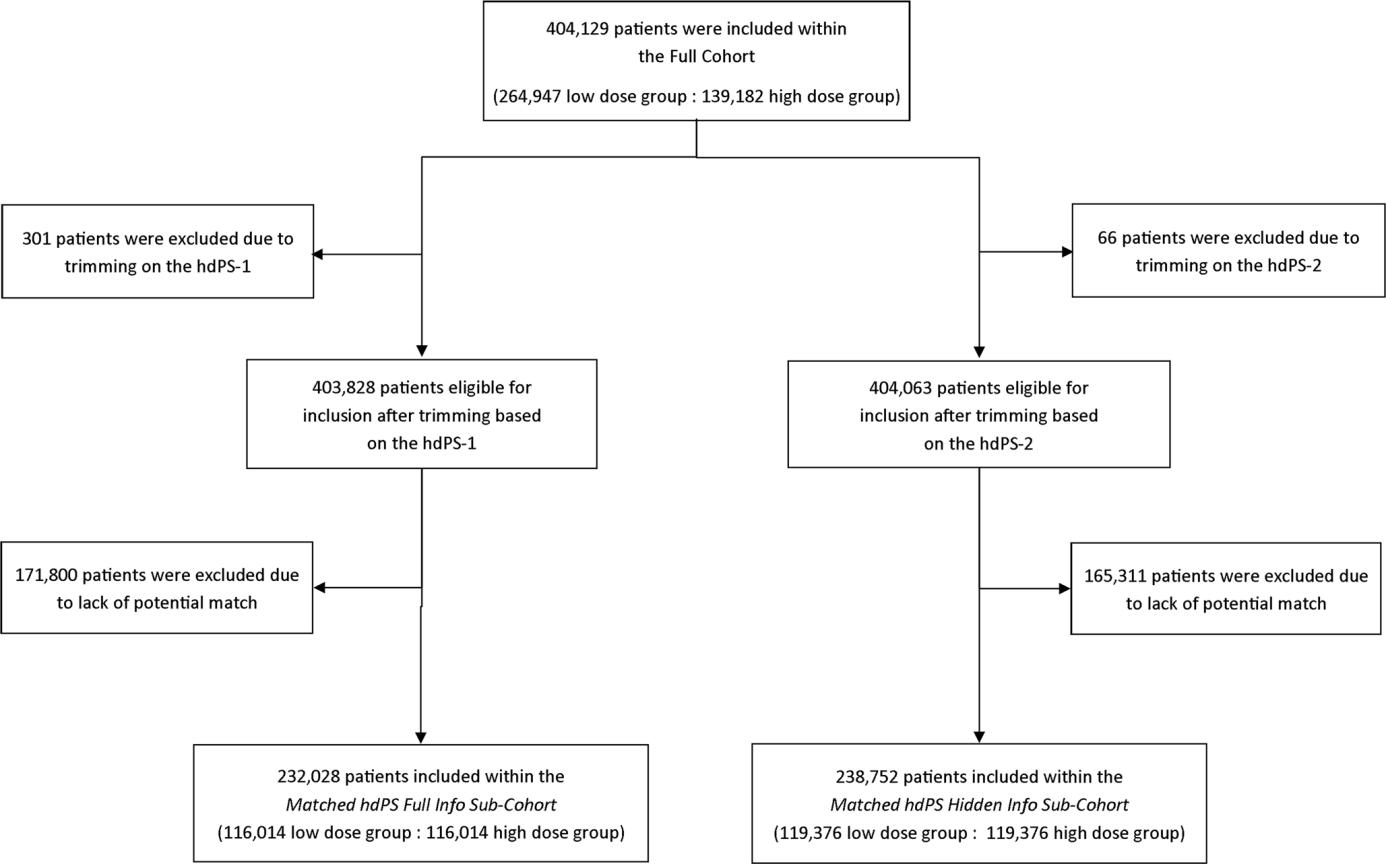
Patient flow-chart within the study. *hdPS* high-dimensional propensity score

Figure 1 shows the flow chart of patients from their inclusion within the *Full Cohort* to their inclusion within the *Matched hdPS Full Info Sub-Cohort* and the *Matched Hidden Info Only Sub-Cohort.*

The *Full Cohort* is comprised of 404,129 patients, 264,947 patients (65.6 %) of which were in the low dose group while the remaining 139,182 patients (34.4 %) were in the high dose group; as mentioned previously, patients in the high dose group were different and overall sicker than those in the low dose group. Specifically, eight of the 18 examined patient characteristics (i.e., sex, ≥5 outpatient medical visits, ≥1 hospitalization, history of myocardial infarction, history of percutaneous coronary intervention, dispensation of beta-blockers, dispensation of angiotensin receptor blockers [ARB], dispensation of angiotensin converting enzyme inhibitors [ACEI]) were unbalanced (ASDD >0.1) within the *Full Cohort* [7].

Of the 404,129 patients included within the *Full Cohort*, none were classified as diabetic at the cohort entry date. Diabetes was identified in 12,978 patients (3.2 %) within the 2 years follow-up.

### Description of the selected data dimensions

Table 1 shows the number of potential covariates, with and without the assessment of recurrence, within each of the 6 data dimensions considered within this study. The 4 data dimensions provided from the RAMQ databases (*n* without assessment of recurrence =2758 [71.6 %], *n* with assessment of recurrence =5011 [81.0 %]) contained a greater number of potential covariates than the 2 data dimensions provided from MED-ECHO database (*n* without assessment of recurrence =1096 [28.4 %], *n* with assessment of recurrence =1174 [19.0 %]).

**Table 1:** Number of covariates available within each data dimension provided from the two Quebec medico-administrative databases

Data dimension	Number of potential covariates available within the data dimension^a^	Number of potential covariates available following the assessment of recurrence of the covariate within the data dimension
RAMQ database		
Outpatient drug dispensations	524	1320
Inpatient and outpatient diagnostic codes	1202	1986
Inpatient and outpatient procedure codes	993	1610
Speciality of the physician	39	95
MED-ECHO database		
Inpatient diagnostic codes	843	915
Inpatient procedure codes	253	259

The *hdPS full info* model was created from the information present within all 6 data dimensions while the *hdPS hidden info* model was limited to the information present within the 2 data dimension provided by MED-ECHO

*MED-ECHO Maintenance et Exploitation des Données pour l’Étude de la Clientèle Hospitalière* ; *RAMQ Régie de l’assurance maladie du Québec*

^a^Any covariate not present within at least 100 patients is excluded by the hdPS algorithm and was therefore not included within this table

### Characteristics of the patients included within the Matched hdPS Full Info Sub-Cohort

Using data contained within all 6 available high-dimensions, we created the *hdPS full info* model which was used to estimate patients’ hdPS-1. Three hundred and one patients (0.0 %) had hdPS-1 located within non-overlapping regions and were excluded from the analysis. Among the remaining 403,828 patients, we matched 116,014 patients (28.7 %) from the high dose group to 116,014 patients (28.7 %) from the low dose group based on their individual hdPS-1; selected patients formed the *Matched hdPS Full Info Sub-Cohort* (Fig. 1).

Patients included within the *Matched hdPS Full Info Sub-Cohort* were on average 64.6 years old (SD 11.2) and 116,688 of them were males (50.3 %) (Table 2). Balance (ASDD <0.1) was obtained in all 18 examined patient characteristics (ASDD ranged from 0.001 to 0.023 with an average of 0.008).

**Table 2:** Demographic characteristics and comorbidity status of the *Matched hdPS Full Info Sub-Cohort* at baseline

	Low dose group *n* (%)	High dose group *n* (%)	Absolute standardized differences
	116,014 (100.0)	116,014 (100.0)	
Age, mean (SD)^a^	64.6 (11.2)	64.6 (11.2)	0.002
Male sex^b^	58,194 (50.2)	58,494 (50.4)	0.005
At least 5 medical outpatient visits^b^	66,453 (57.3)	66,390 (57.2)	0.001
At least 1 hospitalization^c^	28,265 (24.4)	28,604 (24.7)	0.007
Myocardial infarction^c^	7558 (6.5)	7995 (6.9)	0.015
Stroke	3620 (3.1)	3897 (3.4)	0.013
Hypertension	48,268 (41.6)	48,474 (41.8)	0.004
Dyslipidemia	37,486 (32.3)	37,841 (32.6)	0.007
Peripheral vascular disease	2293 (2.0)	2671 (2.3)	0.023
Congestive heart failure	5198 (4.5)	5479 (4.7)	0.012
Coronary artery bypass graft	1670 (1.4)	1661 (1.4)	0.001
Percutaneous coronary intervention^c^	4590 (4.0)	4846 (4.2)	0.011
Dispensation of loop diuretics	7139 (6.2)	7256 (6.3)	0.004
Dispensation of calcium blockers	26,510 (22.9)	26,716 (23.0)	0.004
Dispensation of beta-blockers^b^	33,901 (29.2)	34,389 (29.6)	0.009
Dispensation of angiotensin receptor blockers^b^	20,345 (17.5)	20,876 (18.0)	0.012
Dispensation of angiotensin converting enzyme inhibitors^b^	24,472 (21.1)	25,289 (21.8)	0.017
At least 5 different drugs dispensed	66,600 (57.4)	66,820 (57.6)	0.004

Comorbidity status, drug dispensations, and medical utilization rates were assessed in the year prior to the cohort entry date. Absolute standardized differences are defined as the between group difference as a proportion of the pooled standard deviation of the two groups

^a^At the cohort entry date

^b^Identifies baseline characteristics which had 0.10< ASDD ≤0.20 within the unmatched populations [7]

^c^Identifies baseline characteristics which had ASDD >0.20 within the unmatched populations [7]

### Characteristics of the patients included within the Matched hdPS Hidden Info Sub-Cohort

Using data from the 2 data dimensions selected from the MED-ECHO databases, we created the *hdPS hidden info* model to estimate each patient’s individual hdPS-2. Sixty-six patients (0.0 %) had hdPS-2 located within non-overlapping regions and were excluded from the analysis. Among the remaining 404,063 patients, we matched 119,376 patients (29.5 %) from the high dose group to 119,376 patients (29.5 %) from the low dose group based on their individual hdPS-2; selected patients formed the *Matched hdPS Hidden Info Sub-Cohort* (Fig. 1). About half of the patients included within this sub-cohort were male (*n* = 120,238 [50.4 %]) and the average age was 64.5 years old (SD 11.2) (Table 3). Balance within this sub-cohort was obtained for all 18 examined patient characteristics (ASDD ranged from 0.004 to 0.075 with an average of 0.027), including those which were hidden to the hdPS algorithm (ASDD for the hidden covariates ranged from 0.011 to 0.075 with an average of 0.036).

**Table 3:** Demographic characteristics and comorbidity status of the *matched hdPS hidden info sub-cohort* at baseline

	Low dose group *n* (%)	High dose group *n* (%)	Absolute standardized differences
	119,376 (100.0)	119,376 (100.0)	
Age, mean (SD)^a^	64.5 (11.2)	64.6 (11.1)	0.012
Male sex^b^	59,870 (50.2)	60,368 (50.6)	0.008
At least 5 medical outpatient visits^b,d^	69,706 (58.4)	67,866 (56.9)	0.031
At least 1 hospitalization^c^	28,176 (23.6)	29,679 (24.9)	0.029
Myocardial infarction^c^	7427 (6.2)	8605 (7.2)	0.039
Stroke	3515 (2.9)	3961 (3.3)	0.021
Hypertension	49,608 (41.6)	49,833 (41.7)	0.004
Dyslipidemia	38,734 (32.5)	38,328 (32.1)	0.007
Peripheral vascular disease	2248 (1.9)	2742 (2.3)	0.029
Congestive heart failure	4977 (4.2)	5804 (4.9)	0.033
Coronary artery bypass graft	1550 (1.3)	1717 (1.4)	0.012
Percutaneous coronary intervention^c^	4541 (3.8)	5324 (4.5)	0.033
Dispensation of loop diuretics^d^	6852 (5.7)	7604 (6.4)	0.026
Dispensation of calcium blockers^d^	26,961 (22.6)	27,501 (23.0)	0.011
Dispensation of beta-blockers^b,d^	32,994 (27.6)	37,067 (31.1)	0.075
Dispensation of angiotensin receptor blockers^b,d^	20,479 (17.2)	21,877 (18.3)	0.031
Dispensation of angiotensin converting enzyme inhibitors^b,d^	24,286 (20.3)	26,996 (22.6)	0.055
At least 5 different drugs dispensed^d^	68,169 (57.1)	69,442 (58.2)	0.022

Comorbidity status, drug dispensations, and medical utilization rates were assessed in the year prior to the cohort entry date. Absolute standardized differences are defined as the between group difference as a proportion of the pooled standard deviation of the two groups

^a^At the cohort entry date

^b^Identifies baseline characteristics which had 0.10< ASDD ≤0.20 within the unmatched populations [7]

^c^Identifies baseline characteristics which had ASDD >0.20 within the unmatched populations [7]

^d^Identifies covariates which were hidden to the hdPS algorithm within the *hdPS hidden info* model

### Relative performance of the two matched sub-cohorts

**Fig. 2:**
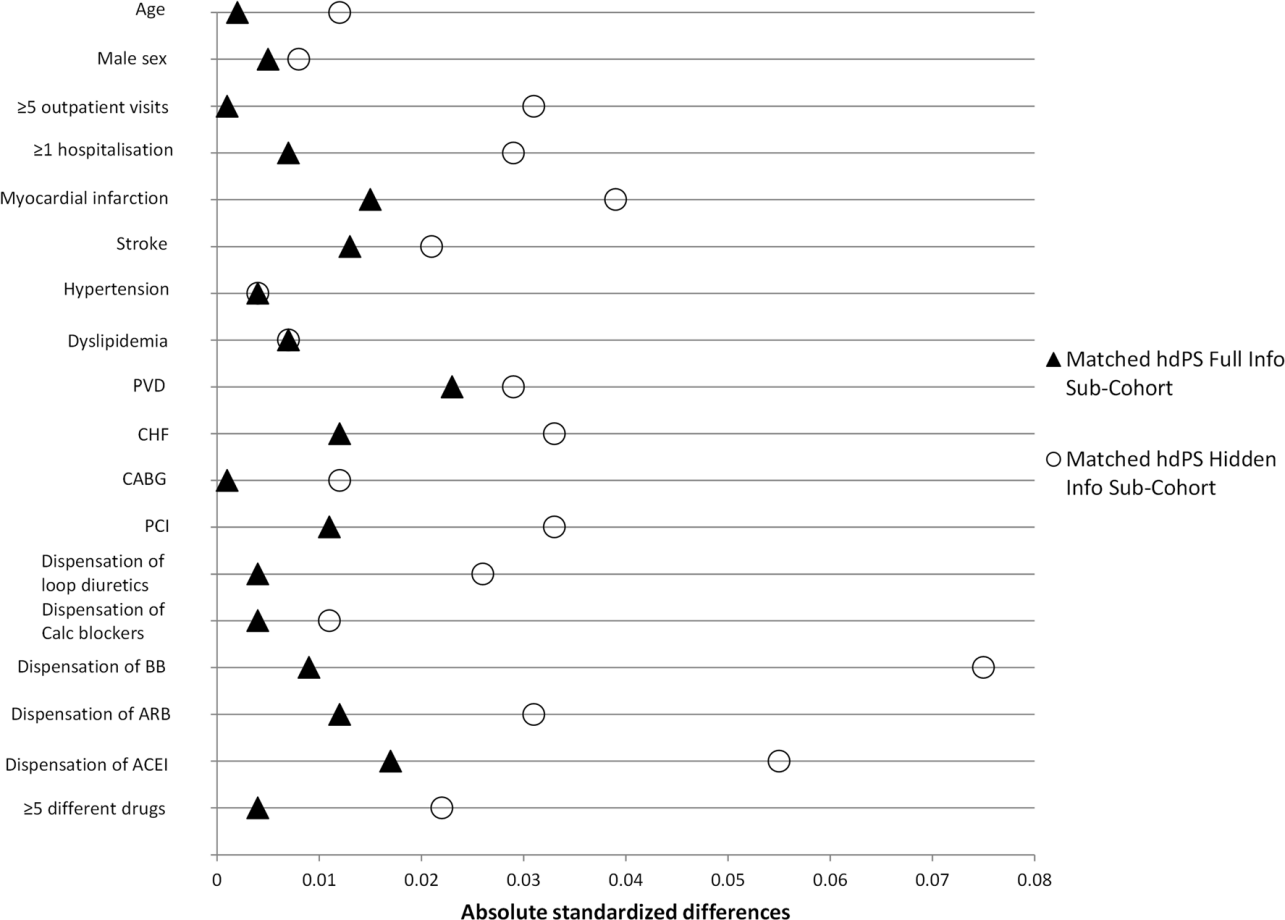
Head-to-head comparison of the absolute standardized differences obtained within the two matched sub-cohorts. *ACEI* angiotensin converting enzyme inhibitors; *ARB* angiotensin receptor blockers; *BB* beta-blockers; *CABG* coronary artery bypass graft; *Calc blockers* calcium blockers; *CHF* congestive heart failure; *hdPS* high-dimensional propensity score; *PCI* percutaneous coronary intervention; *PVD* peripheral vascular disease; Absolute standardized differences <0.1 are assumed to indicate balance; all 18 patient characteristics were considered to be balanced within the two sub-cohorts

ASDD obtained within both matched sub-cohorts are shown within Fig. 2. The *Matched hdPS Full Info Sub-Cohort* was shown to achieve better balance on 16 of the 18 examined patient characteristics, the two remaining characteristics were equally balanced within both matched sub-cohorts.

## Discussion

Our results show that matching on the *hdPS hidden info* model achieved balance on all 18 examined patient characteristics. This result shows that the hdPS algorithm was able to adjust for imbalance regarding patient characteristics, some of which were unavailable to the hdPS algorithm. Among these, some were very important variables regarding outpatient medical visits and drug dispensations which can be highly associated with both the choice of treatment and the risk of diabetes.

As expected the hdPS algorithm had access to a greater number of potential covariates to build the *hdPS full info* model (*n* = 3854 potential covariates) than when building the *hdPS hidden info* model (*n* = 1096 [28.4 %] potential covariates). This difference implied that 431 (86.2 %) covariates selected within the *hdPS full info* model were no longer available for selection and had to be replaced by the algorithm when it built the *hdPS hidden info* model.

The main strength of our study is that it provides support to the claim that the hdPS is able to adjust for at least some unmeasured confounders. In their original paper, Schneeweiss and colleagues [5] hinted that some of the covariates selected by the hdPS algorithm may not be direct confounders but may actually be proxies of unmeasured confounders. Although adjusting for a perfect proxy of an unmeasured confounder is equivalent to directly adjusting for this confounder [28], it remained unclear if the hdPS could truly adjust for a confounder not present within the examined database. Four important known confounders (i.e., ≥5 medical outpatient visits, dispensation of beta-blockers, dispensation of ARB, and dispensation of ACEI; all shown to be unbalanced within the *full cohort*) [7] were not available to the *hdPS hidden info* model. Our results show that this model was able to achieve balance within all examined patient characteristics, including the four previously mentioned (Table 3). Such a result is of significant value since the PS technique may not adjust for variables not included within the PS model [29]. However, we were unable to identify which covariates selected by the hdPS algorithm were used as proxies for these four confounders.

Our study has limitations. *First*, since our study shows that hdPS was able to control for measured confounders which were unavailable to the hdPS algorithm in the restricted data setting, it is reasonable to think that the algorithm is also able to control for some unmeasured confounders. Of note, the ability of hdPS to control for unmeasured confounders may be specific to these covariates/databases and to this specific population and may not be true in other settings.

*Second*, we only examined a limited number of patient characteristics. Although balance was achieved within both sub-cohorts regarding all 18 examined patient characteristics, we cannot guarantee that this balance would be achieved in other patient characteristics or in other unmeasured confounders.

*Finally*, we did not examine the relative performance of the two matched sub-cohorts in regards to the measure of association which would have been obtained in an eventual etiological study. To do so would require the existence of a “gold standard”, providing the nature and magnitude of the “true” association, to which we could compare our results [2–5]. Despite this fact, we consider that the quality of the match is a good marker of the performance of the hdPS method within this study since only this approach could illustrate that the hdPS method truly adjusted for the seven hidden confounders [7, 17–21].

In conclusion, our results show that, within the confines of our study, the hdPS was able to adequately adjust for confounders which were hidden to the algorithm. Such results support the claim that the hdPS can adjust for at least some unmeasured confounders and further support its use in future observational studies.

## References

[1] Black CM, Tadrous M, Cadarette SM (2013) Diffusion of methodological innovation in pharmacoepidemiology: high-dimensional propensity score co-authorship network analysis. Paper presented at the CAPT, Toronto,

[2] Garbe E, Kloss S, Suling M, Pigeot I, Schneeweiss S (2012). High-dimensional versus conventional propensity scores in a comparative effectiveness study of coxibs and reduced upper gastrointestinal complications. Eur J Clin Pharmacol.

[3] Polinski JM, Schneeweiss S, Glynn RJ, Lii J, Rassen JA (2012). Confronting “confounding by health system use” in Medicare Part D: comparative effectiveness of propensity score approaches to confounding adjustment. Pharmacoepidemiol Drug Saf.

[4] Rassen JA, Glynn RJ, Brookhart MA, Schneeweiss S (2011). Covariate selection in high-dimensional propensity score analyses of treatment effects in small samples. Am J Epidemiol.

[5] Schneeweiss S, Rassen JA, Glynn RJ, Avorn J, Mogun H, Brookhart MA (2009). High-dimensional propensity score adjustment in studies of treatment effects using health care claims data. Epidemiology.

[6] Franklin JM, Schneeweiss S, Polinski JM, Rassen JA (2014). Plasmode simulation for the evaluation of pharmacoepidemiologic methods in complex healthcare databases. Comput Stat Data Anal.

[7] Guertin JR, Rahme E, Dormuth CR, LeLorier J (2016). Head to head comparison of the propensity score and the high-dimensional propensity score matching methods. BMC Med Res Methodol.

[8] Gouvernement du Québec (2014) Banque de données ministérielles MED-ÉCHO. http://www.ramq.gouv.qc.ca/fr/donnees-statistiques/sur-demande/donnees-msss/Pages/med-echo.aspx. Accessed 2014–08-04

[9] Gouvernement du Québec (2014) Services pharmaceutiques. http://www.ramq.gouv.qc.ca/fr/donnees-statistiques/sur-demande/donnees-regie/Pages/services-pharmaceutiques.aspx. Accessed 2014–08-04

[10] Gouvernement du Québec (2014) Services médicaux rémunérés à l'acte. http://www.ramq.gouv.qc.ca/fr/donnees-statistiques/sur-demande/donnees-regie/Pages/services-medicaux-remuneres-acte.aspx. Accessed 2014–08-04

[11] Carter AA, Gomes T, Camacho X, Juurlink DN, Shah BR, Mamdani MM (2013). Risk of incident diabetes among patients treated with statins: population based study. BMJ.

[12] Dormuth CR, Filion KB, Paterson JM, James MT, Teare GF, Raymond CB, Rahme E, Tamim H, Lipscombe L (2014). Higher potency statins and the risk of new diabetes: multicentre, observational study of administrative databases. BMJ.

[13] Ko DT, Wijeysundera HC, Jackevicius CA, Yousef A, Wang J, Tu JV (2013). Diabetes and cardiovascular events in older myocardial infarction patients prescribed intensive-dose and moderate-dose statins. Circ Cardiovasc Qual Outcomes.

[14] Preiss D, Seshasai SR, Welsh P, Murphy SA, Ho JE, Waters DD, DeMicco DA, Barter P, Cannon CP, Sabatine MS, Braunwald E, Kastelein JJ, de Lemos JA, Blazing MA, Pedersen TR, Tikkanen MJ, Sattar N, Ray KK (2011). Risk of incident diabetes with intensive-dose compared with moderate-dose statin therapy: a meta-analysis. JAMA.

[15] Wang KL, Liu CJ, Chao TF, Huang CM, Wu CH, Chen SJ, Chen TJ, Lin SJ, Chiang CE (2012). Statins, risk of diabetes, and implications on outcomes in the general population. J Am Coll Cardiol.

[16] Zaharan NL, Williams D, Bennett K (2013). Statins and risk of treated incident diabetes in a primary care population. Br J Clin Pharmacol.

[17] Austin PC (2009). The relative ability of different propensity score methods to balance measured covariates between treated and untreated subjects in observational studies. Medical decision making : an international journal of the Society for Medical Decision Making.

[18] Ali MS, Groenwold RH, Pestman WR, Belitser SV, Roes KC, Hoes AW, de Boer A, Klungel OH (2014). Propensity score balance measures in pharmacoepidemiology: a simulation study. Pharmacoepidemiol Drug Saf.

[19] Austin PC (2009). Balance diagnostics for comparing the distribution of baseline covariates between treatment groups in propensity-score matched samples. Stat Med.

[20] Belitser SV, Martens EP, Pestman WR, Groenwold RHH, de Boer A, Klungel OH (2011). Measuring balance and model selection in propensity score methods. Pharmacoepidemiol Drug Saf.

[21] Mamdani M, Sykora K, Li P, Normand SL, Streiner DL, Austin PC, Rochon PA, Anderson GM (2005). Reader's guide to critical appraisal of cohort studies: 2. Assessing potential for confounding. BMJ.

[22] Pharmacoepidemiology Toolbox. (2011) Brigham and Women's Hospital. http://www.drugepi.org/dope-downloads/. Accessed 2014–08-04

[23] Rosenbaum PR, Rubin DB (1985). The bias due to incomplete matching. Biometrics.

[24] Sturmer T, Joshi M, Glynn RJ, Avorn J, Rothman KJ, Schneeweiss S (2006). A review of the application of propensity score methods yielded increasing use, advantages in specific settings, but not substantially different estimates compared with conventional multivariable methods. J Clin Epidemiol.

[25] Sturmer T, Rothman KJ, Avorn J, Glynn RJ (2010). Treatment effects in the presence of unmeasured confounding: dealing with observations in the tails of the propensity score distribution—a simulation study. Am J Epidemiol.

[26] Austin PC (2011). Optimal caliper widths for propensity-score matching when estimating differences in means and differences in proportions in observational studies. Pharm Stat.

[27] Austin PC (2011). An introduction to propensity score methods for reducing the effects of confounding in observational studies. Multivar Behav Res.

[28] Woolridge JM (2001). Econometric analysis of cross section and panel data.

[29] Brooks JM, Ohsfeldt RL (2012) Squeezing the balloon: propensity scores and unmeasured covariate balance. Health Services Research:1–21. 10.1111/1475-6773.12020 PMC3725536 23216471

